# Accuracy-Risk Trade-Off Due to Social Learning in Crowd-Sourced Financial Predictions

**DOI:** 10.3390/e23070801

**Published:** 2021-06-24

**Authors:** Dhaval Adjodah, Yan Leng, Shi Kai Chong, P. M. Krafft, Esteban Moro, Alex Pentland

**Affiliations:** 1 Media Lab, Massachusetts Institute of Technology, Cambridge, MA 02139, USA; cshikai@mit.edu; 2 McCombs School of Business, The University of Texas at Austin, Austin, TX 78712, USA; yleng@mit.edu; 3 Oxford Internet Institute, University of Oxford, Oxford OX1 2JD, UK; pkrafft@mit.edu; 4 Departamento de Matemáticas & GISC, Universidad Carlos III de Madrid, 28911 Leganes, Spain; emoro@mit.edu

**Keywords:** crowd-sourcing, wisdom of the crowd, social learning, Bayesian models, risk

## Abstract

A critical question relevant to the increasing importance of crowd-sourced-based finance is how to optimize collective information processing and decision-making. Here, we investigate an often under-studied aspect of the performance of online traders: beyond focusing on just accuracy, what gives rise to the trade-off between risk and accuracy at the collective level? Answers to this question will lead to designing and deploying more effective crowd-sourced financial platforms and to minimizing issues stemming from risk such as implied volatility. To investigate this trade-off, we conducted a large online Wisdom of the Crowd study where 2037 participants predicted the prices of real financial assets (S&P 500, WTI Oil and Gold prices). Using the data collected, we modeled the belief update process of participants using models inspired by Bayesian models of cognition. We show that subsets of predictions chosen based on their belief update strategies lie on a Pareto frontier between accuracy and risk, mediated by social learning. We also observe that social learning led to superior accuracy during one of our rounds that occurred during the high market uncertainty of the Brexit vote.

## 1. Introduction

Distributed financial platforms are on the rise, ranging from Decentralized Autonomous Organizations [1], crowd-sourced prediction systems [2] to the very recent events during which retail investors self-organized using social media and drove up asset and derivative prices [3,4]. In this work, we investigate how financial agents process information from one another and predict-individually and collectively—the future prices of real assets. Specifically, we are interested in understanding the computational models they use to update their beliefs after information exposure and how different social vs. non-social belief update strategies lead to trade-offs in prediction performance.

Here, we expand the typical definition of performance for collective prediction to include the concept of risk. Typically, the prediction performance of collectives and swarms is measured mostly by the accuracy of the group over collections of tasks [5,6,7]. However, it has been shown theoretically [8,9] and observed in a variety of applications [10,11] that there is a fundamental trade-off between prediction accuracy (average error) and prediction risk (variance of error).

This means that for any prediction system, risk will always be present, and that maximizing accuracy will come at the expense of increased risk. Hence, the performance of the system will always exist within a pre-defined Pareto frontier [12,13] which is the curve containing all possible system performance parametrizations (here, pairs of possible accuracy and risk values). Therefore, a platform designer will need to make trade-offs between risk and accuracy and cannot achieve arbitrarily combinations of risk and accuracy. Treating risk and accuracy as equally important for prediction is standard in statistical  [8,9,10] and financial [14,15,16] forecasting applications and literature because it allows for prediction systems to be calibrated and deployed with regard to specific accuracy and risk profiles  [17,18,19,20,21].

However, characterizing the performance of crowd-based prediction systems regarding both accuracy and risk is not common and such a Pareto frontier has not been observed in crowd-sourced financial asset price prediction. We are therefore interested in investigating if a Pareto frontier exists and what the causes are behind this trade-off. From the perspective of crowd-sourced financial platform designers, understanding the trade-off between accuracy and risk and how to select subsets of predictions that achieve a certain accuracy and risk is useful to fit a required risk profile. This, in turn, allows for more sophisticated and versatile applications of crowd-sourced predictions such as hedging risks over portfolios of prediction tasks.

To test our hypothesis that a Pareto frontier exists between risk and accuracy and that it is mediated by social learning, we designed our collective prediction experiments as a series of Wisdom of the Crowd (WoC) tasks. For background, the Wisdom of the Crowd  [22,23] is a popular domain within the collective intelligence literature where participants (the ‘crowd’) are asked to make predictions of a certain quantity, such as the future price of an asset on the stock market [24] or the caloric content of food items [25]. Prior work in the WoC literature [25,26,27] has focused on maximizing the average accuracy of collectives with little regard to the risk of the predictions.

The structure of this paper is as follows: we do a short literature review of the connections of this work to research on collective intelligence and the accuracy-risk trade-off in Section 2. We discuss our materials and methods (experimental design, data collection, and modeling and estimation) in Section 3. We present our results (belief update modeling, accuracy-risk trade-off and prediction under high uncertainty during Brexit) in Section 4. We discuss the implications and limitations of our work in Section 5.

### Contributions

Our work makes the following novel contributions:

- We present an experimental procedure where we exposed 2037 participants to social and non-social information during 7 independent rounds of predicting financial asset prices (S&P 500, gold and WTI Oil). We collected 4634 prediction sets which include participants’ predictions before and after information exposure, as well as the information they were exposed to. We are releasing this data here.
- Using computational models inspired by Bayesian models of cognition [28,29] to investigate the belief update strategy of participants, we observe that a simple model that approximates the likelihood (evidence) to be a unimodal Gaussian beats a more complex Monte Carlo approach. This suggests that our participants exhibit the attribute substitution heuristic of human decision-making [30], whereby a complicated problem is solved by approximating it with a simpler, less accurate model.
- We observe that participants prefer to learn from social information rather than from non-social information, another interesting information processing heuristic.
- Our main contribution: we observe a Pareto frontier between accuracy and risk. As the average accuracy of the crowd over the different prediction rounds increases, so does the risk in the crowd’s predictive accuracy. We further observe that this trade-off is mediated by the amount of social learning i.e., the extent to which participants pay attention to each other’s judgments.
- We deployed one of our prediction tasks just before the Brexit vote during which there was a great deal of market uncertainty [31], and we observe that during such uncertain times social learning leads to higher accuracy.

These results are not only important for the practical deployment of distributed financial prediction platforms but also expand our understanding of how financial agents process information and make distributed predictions.

## 2. Related Work

### 2.1. Collective Intelligence and Social Learning

There is a rich literature on how decentralized information processing, learning and decision-making affects the performance of collectives and swarms [32,33,34,35,36]. Here, we focus on how platforms can be designed for people to make predictions with high performance, which is a central question for the Wisdom of the Crowd [22,23,37].

It has been shown that the temporal influence and mutual information dynamics between individuals can have a strong effect on crowd collective performance. On the one hand, prior work has shown that exposure to social information can lead to degraded performance in aggregate guesses [26,37,38]. For example, increasing the strength of social influence has been shown to increase inequality [39]. Selecting the predictions of people who are resistant to social influence has been shown to have improved collective accuracy  [27]. The influence of influential peers has been theoretically shown to prevent the group from converging on the true estimate [26], and exposure to the confidence levels of others has been shown to influence people to change their predictions for the worse [40].

On the other hand, social learning has also been shown to lead to groups outperforming their best individuals when they work separately [41] and a collective intelligence factor has been shown to predict team performance better than the maximum intelligence of members of the team [35]. Similarly, human-inspired social communication between agents has been shown to improve collective performance in optimization algorithms [5,42].

Therefore, the role of social learning in collective performance is still being understood. Our contribution to this line of research is that a more complete characterization of performance in terms of not just accuracy but also risk provides avenues for future work towards reconciling the disagreements as to the role of social influence on performance. This is especially important due to the already existing strong social components in many crowd-sourcing platforms and applications [43,44,45,46,47,48] that could be harnessed more effectively for performance improvement.

### 2.2. Accuracy-Risk Trade-Off

Previous work has investigated several avenues to optimize the accuracy of the crowd such as by recalibrating predictions against systematic biases of individuals [26] and selecting participants who are resistant to social influence [27]. Additionally, rewiring the network topology of information-sharing between subjects [25,41], and optimally allocating tasks to individuals [49] has improved collective accuracy. However, these studies focused on accuracy with little regard to risk. There is a rising movement to go beyond accuracy and to fully characterize performance—at the individual and the collective level—in terms of both accuracy and risk. Some call this emerging line of work going beyond the ‘bias bias (In the statistics literature, bias is another name for accuracy. This movement suggests that research should go beyond its current focus on just bias and study risk).

At the individual level, there is increasing evidence that people preferentially optimize for risk instead of accuracy in a variety of domains [50]. Cognitively, people have been observed to manifest decision heuristics [51] to be conservative in the face of uncertainty [52,53]. For example, rice farmers have been observed not to adopt significant harvest improvement technology because of the risk of it failing once and causing significant family ruin [54]. Evolutionarily, risk aversion has been shown to emerge when rare events have a large impact on individual fitness [52]. Furthermore, in a meta-study of 105 forecasting papers, 102 of them support prioritizing for lower risk to achieve higher overall performance [55]. At the collective level, there is limited work regarding the characterization of the performance of collectives and swarms in terms of both accuracy and risk although there is a large literature on other related trade-offs such as between speed and accuracy [56,57,58,59,60].

From a system design perspective, crowd-sourcing platform designers should characterize their performance in terms of both accuracy and risk due to theoretical results [8,9] and observations in applications [10,11] that the performance of any prediction system is subject to a fundamental trade-off between accuracy and risk. This is especially important in our domain of predicting financial asset prices as risk is already known to have negative effects on the efficiency of markets such as through the phenomenon of implied volatility [61].

## 3. Materials and Methods

### 3.1. Experimental Design

To test our hypothesis that a Pareto frontier exists between risk and accuracy—i.e., that there is a trade-off between risk and accuracy of prediction across several prediction rounds—and that it is mediated by social learning, we need a dataset with the following requirements:

- Predictions are made of complex and difficult-to-predict phenomena so that our results are applicable to the real-world platform applications.
- Predictions are made over many independent prediction rounds so that the risk of the crowd over these different tasks can be estimated.
- A ground-truth is needed against which we can compare our dataset to judge the external validity of individual and collective performance metric.
- The social and non-social information each participant was exposed to after their initial pre-exposure prediction is recorded so that we can later model how different types of information influenced them in updating their belief into their post-exposure prediction.

Given the above requirements, we designed the experimental procedure as detailed below: we recruited a total of 2037 participants over seven prediction rounds to predict the future prices of financial assets (the S&P 500, WTI Oil, and gold prices) during seven separate consecutive 3-week rounds over the span of 6 months, resulting in 9268 predictions (i.e., 4634 prediction pairs or sets). We focused on predicting financial prices as doing so is a hard prediction problem [62,63]. Our participants were mid-career financial professionals with years of financial experience. Our participants consented to their data being used in this study and we obtained prior IRB approval. One of our rounds of prediction happened to end the day of the Brexit vote, which means that we have prediction data during a particularly volatile market period [31] as described in Supplementary Section A.5.

During each round, participants made a prediction of the same asset’s closing price for the same final day of the round. We use the round’s last day’s closing market price as our measure of ground-truth. We carefully instrumented the social and non-social information that our participants were exposed to, and collected their predictions before and after exposure to this information. We also deployed one of our rounds during a high uncertainty period to understand if variance reduction strategies allow the crowd to be resistant to risk.

We did not opt for an A/B testing experimental design [64]—where we would have split participants and shown each group either the social information or the historical price time series—because we wanted participants to naturally choose whichever source of information to use to update their belief. This was an important experimental design choice as we wanted to understand, as close as possible to in-situ how people update their beliefs in the real-world where they are already exposed to both their peers’ beliefs and to price history information, such as through financial news. Our design is in contrast to previous work where the experiments were deployed within a carefully controlled laboratory set-up as in prior work [25,37,40].

### 3.2. Data Collection

**Figure 1:**
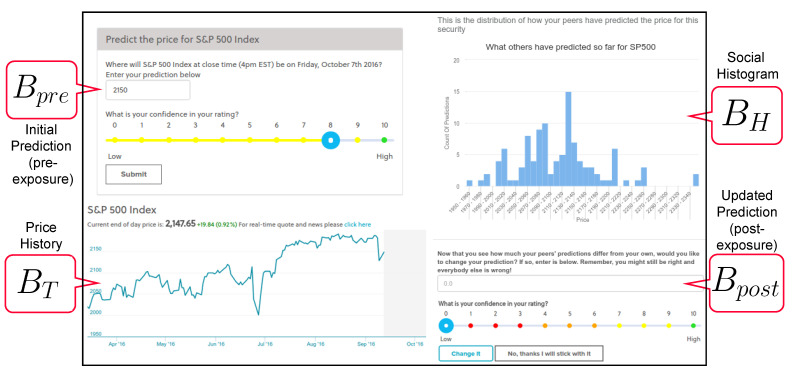
An annotated screenshot of how data were collected: the pre-exposure prediction $B_{pre}$ is shown first, followed by the social histogram $B_{H}$ and the price history $B_{T}$. Finally, the updated prediction $B_{post}$ is collected. The ground-truth of the asset’s final closing price will be *V* (not shown here, realized at the end of the round).

As shown in the screenshot of the user interface in Figure 1, we designed the data collection process as follows: every time a participant makes a prediction of an asset’s future price through our platform, the following prediction set comprising $B_{pre},B_{H},B_{T}$ and $B_{post}$ is collected:

- A “pre-exposure” belief prediction $B_{pre}$, which is independent of *both* social information and price history. For example, a participant might show-up on the platform and predict that the closing price of the S&P 500 to be 2001 on 24 June 2016.
- The predictions $B_{H}$ within the social information histogram shown to each participant after each initial prediction. Additionally, we display a 6-month time series of the asset’s price $B_{T}$ up to this point.
- The revised “post-exposure” prediction $B_{post}$. For example, after seeing the social histogram and asset price history, a participant might update their belief to 2201. Since the real price (the ground-truth *V*) ended up being 2037.41, this participant became more accurate after information exposure (they went from 2001 to 2201).

Overall, we ensure that the “pre-exposure” prediction is made before any social information and price history is shown. We present a unique histogram for every new prediction (as it is built using past predictions up to this point), as well as a unique price history time series (as it shows the 6-month price data up to the time of prediction). We require all participants to make a post-exposure prediction even if they decide to keep it at the pre-exposure level.

### 3.3. Modeling and Estimation

Using the data collected in the live experiments, we want to test our hypothesis that a Pareto frontier exists between risk and accuracy and that it is mediated by social learning. In this section, we describe all the modeling and estimation steps required to investigate our hypothesis:

- In Section 3.3.1, we describe how we model individual belief update: how a participant updates their prediction from a pre-exposure belief to a post-exposure prediction using a variety of models that are either Monte Carlo methods or simpler approximate methods inspired by Bayesian models of cognition [28,29]. This allows us to understand how participants update their belief after information exposure.
- In Section 3.3.2, using the models described earlier, we detail how to estimate the relative amount of social vs. non-social learning for each prediction to understand how much social vs. non-social data were factored into a prediction’s belief update. We then introduce our methodology for selecting predictions based on the estimated amount of social vs. non-social learning. This allows us to make aggregate predictions—at the platform level—based on a pre-specified amount of social learning.
- In Section 3.3.3, we detail how the accuracy and risk—at the platform level—of selected subsets are measured, and how they are used to investigate whether a Pareto trade-off exists between accuracy and risk and whether it is mediated by the relative amount of social vs. non-social learning.

#### 3.3.1. Modeling Belief Updates

Using formalism inspired by Bayesian models of cognition [29], we can model the 4634 prediction sets collected over many rounds, at a high level, as a Bayesian update. To use this formalism, we need to select a prior distribution for each individual’s belief before exposure to any information and a likelihood (evidence) distribution to model the data participants are exposed to. Additionally, a sampling or approximate method is required to use the prior and evidence to compute the posterior (updated belief after information exposure) distribution. Here, we describe the modeling assumptions and procedure at a high level, and detail more thoroughly our modeling assumptions and present our derivations in Supplementary Section A.3.

Fundamentally, we are interested in how participants predict an asset’s future price (ground-truth) *V* based on the information we expose them to. The choice of the prior distribution is straightforward: $P_{prior}\left(V\right)\approx P\left(B_{pre}\right)$, the distribution of belief of an individual before they are exposed to any information. We discuss in our model derivation (Supplementary Section A.3) how, when needed, we approximate the full distribution $P(B_{pre})$ since we obtain only one sample, $B_{pre}$, for each participant and cannot observe the full distribution $P(B_{pre})$.

After participants input their pre-exposure belief $B_{pre}$, there are two main likelihood (evidence) distributions participants employ: they are exposed to the assets’ price history $B_{T}$, giving us $P_{likelihood}\left(V\right)\approx P\left(B_{T}\right)$, or analogously, the social histogram $B_{H}$, giving us $P_{likelihood}\left(V\right)\approx P\left(B_{H}\right)$. In the modeling stage here, we assume that participants used these two likelihood distributions separately to update their beliefs, but we relax this assumption in the estimation stage next where we estimate the relative amount of social vs. non-social learning for each prediction. We detail in Supplementary Section A.3 how likelihood distributions are built from the information that participants are exposed to. In Supplementary Section A.2, we formally detail how we transform the price history into a cognitively accurate ‘rates histogram’ using price momentum. As a summary, because it has been shown that people process time series as a distribution of changes as opposed to a distribution of the quantity itself [65,66,67], we convert the price history time series into a histogram of daily changes (slopes) in prices which is used for both the simple Gaussian models and the numerical models for price prediction.

Given the prior and likelihood, the *modeled* posterior prediction $P_{posterior}\left(V\right)$, can, therefore, be approximated as $P_{posterior}\left(V\right)\propto P\left(B_{H}\right)\cdot P\left(B_{pre}\right)$ in the case of exposure to social information, and $P_{posterior}\left(V\right)\propto P\left(B_{T}\right)\cdot P\left(B_{pre}\right)$ when participants are exposed to the past price history. We do not make any other assumptions in terms of what data to use to approximate the likelihood and prior distributions. Given these distributions, the question is then how to compute the posterior (updated) belief of an individual.

Although we focus on Bayesian models in this work, we include one popular model commonly used as a benchmark in the literature, the DeGroot model [68]. In this model, an individual updates their belief as the weighted average belief of their peers where weights can be, for example, trust values of the individual for their peers. Here we set the weights (trust values) equal for all peers, as we have no data to estimate these weights, and therefore assume a uniform prior.

Although the space of possible distributions and posterior computation approaches is very large, we focus here on using two simple, interpretable, and theoretically motivated approaches from prior work [28]. We either use Gaussian (normal) conjugate distributions to approximate priors and likelihoods due to strong evidence of their ubiquity as Bayesian models of cognition [29], or use a full Monte Carlo numerical sampling approach to calculate the posterior from the actual distributions of prices that participants were exposed to. We leave to future work the exploration of richer distributions and approaches to modeling belief update as it is beyond the scope of this study.

#### 3.3.2. Subsetting Predictions Based on Social Learning

Based on how participants update their belief, we would like to select subsets of predictions based on whether they were more likely updated using social or non-social information. This approach of using characteristics of how predictions are updated is standard in the Wisdom of the Crowd literature. For example, prior work has estimated resistance to social influence [27] and influenceability in revising judgments after seeing the opinion of others [69,70], and used them to improve collective performance. No prior work has investigated investigating if the modeling of belief update strategies could be leveraged for improved collective performance.

Using the previously modeled posteriors, we can *estimate* how much of each information source—social information and price history—each participant used to update their belief by comparing the residual errors of models using either only social information or only price history as likelihood. As will be introduced in the Section 4, although we explored many models of belief update, the simple conjugate Gaussian models model best how participants update their belief. This is in line with previous research showing that although simple, they are highly accurate models of mental estimation in a variety of domains [28].

Therefore, for the purposes of selecting subsets of prediction based on their relative amount of social vs. non-social learning, we choose to focus on the GaussianSocial and GaussianPrice. These models assume the likelihood (evidence) data distribution to be built, respectively, from the social information and price history participants are exposed to.

**Figure 2:**
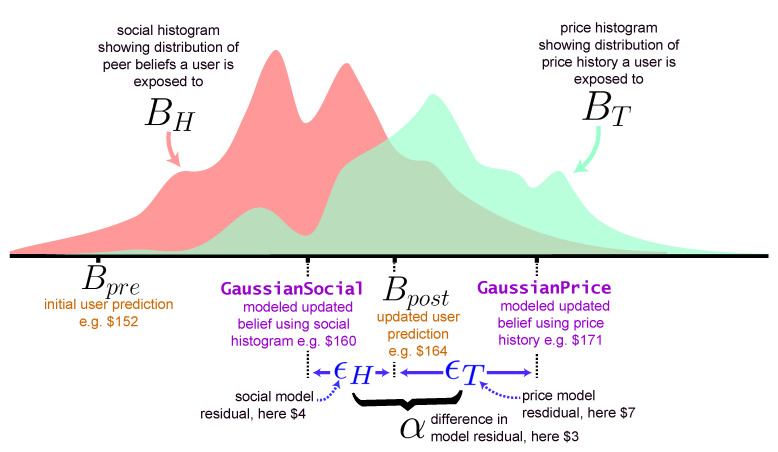
An example belief update: for each prediction set, a participant updates their belief from the pre-exposure prediction $B_{pre}$ to the updated prediction $B_{post}$ by either learning from the social histogram $B_{H}$ and/or the price history $B_{T}$. $\epsilon_{H}$ is the residual between the *modeled* updated prediction GaussianSocial and the participant’s updated prediction $B_{post}$; $\epsilon_{T}$ is the residual between GaussianPrice and $B_{post}$. α is the difference between $\epsilon_{T}$ and $\epsilon_{H}$.

Our approach is illustrated in Figure 2: using the prediction of the models Gaussian Social and GaussianPrice, we calculate a residual $\epsilon_{H}$ for when updating belief using social information $B_{H}$ and a residual $\epsilon_{T}$ when updating from the price history $B_{T}$, as $\epsilon_{H}=\frac{|\mathrm{GaussianSocial}-B_{post}|}{B_{post}}$ and $\epsilon_{T}=\frac{|\mathrm{GaussianPrice}-B_{post}|}{B_{post}}$ respectively. We define $\alpha =\epsilon_{T}-\epsilon_{H}$, and we use it to measure how likely a participant used each source of information to update their prediction. For example, for a prediction set $\left[B_{pre},B_{H},B_{T},B_{post}\right]$ if α>0 (i.e., $\epsilon_{T}>\epsilon_{H}$), this means that this prediction set is better modeled using the social histogram of peer’s belief $B_{H}$ instead of the price history $B_{T}$.

Using α, which we re-scale to be in the interval [−1,1] for each round, we can select a subset $S_{\alpha_{s}}$ of the prediction sets such that the α of these prediction sets lie in the range $0\leq \alpha <\alpha_{s}$ (or $\alpha_{s}<\alpha \leq 0$ when $\alpha_{s}<0$). $\alpha_{s}$ is the one-sided boundary we will vary to measure how much more likely a participant updated their belief from the social information instead of the price history. For example, the higher $\alpha_{s}$ is, the more likely a prediction set is better modeled using the social histogram of peer’s belief $B_{H}$ instead of the price history $B_{T}$.

It is important to note that the residuals we use to select subsets are belief update model residuals (between the observed updated belief and the predicted modeled updated belief) which are uncorrelated with the crowd residual (between the crowd’s aggregate prediction and the ground-truth).

#### 3.3.3. Evaluating Improvement of Subsets

Our hypothesis is that a Pareto frontier exists between risk and accuracy and that this trade-off is mediated by the relative amount of social vs. non-social learning.

To test this hypothesis, we investigate how the accuracy and variance of subsets $S_{\alpha_{s}}$ of predictions selected using $\alpha_{s}$ (a measure of the relative amount of social vs non-social learning) compares to the current standard Wisdom of the Crowd approach whereby all predictions are used.

From the perspective of platform designers who want to be able to select predictions based on required levels of accuracy or risk (e.g., to fit a certain portfolio of risk), it is important to measure improvement of subsets relative to the full collection of predictions. This is because, currently, platform designers only have access to one global measure of risk and accuracy—that of the whole set of predictions (when there is no subset filtering). To demonstrate that selecting subsets of predictions can lead to significant *improvements* in accuracy and risk, we therefore need to calculate these improvements.

We therefore define improvement $I^{S_{\alpha_{s}}}$ as the absolute difference between the error $e_{S_{\alpha_{s}}}$ when using a subset $S_{\alpha_{s}}$ compared to the error $e_{S_{all}}$ when using the full set of predictions $S_{all}$, the Wisdom of the Crowd, where $S_{all}$ is defined as the full subset over all predictions using −1≤α≤1.

The error $e_{i,S_{\alpha_{s}}}$ over all predictions $j\in S_{\alpha_{s}}$ for an estimated amount $\alpha_{s}$ of relative social vs. non-social information during experiment round *i* is defined as $\frac{|\sum_{j\in S_{\alpha_{s}}}\left[B_{post,j}\right]-V_{i}|}{V_{i}}$. To allow for estimation uncertainty over the improvement in accuracy and risk of subsets, we use 100 bootstraps with replacement. This procedure is formally described in Supplementary Section A.3.4.

We use an analogous approach to estimate the risk of the platform by calculating the standard deviation instead of the mean of the improvements over experiment rounds. This measures the risk for platform designers to estimate, over a basket of prediction rounds, what is the variance of improvements over this basket. This is the same as understanding the variance of error of a statistical prediction model (e.g., machine learning model) such that we can calibrate both the accuracy and variance of the model over a portfolio of predictions.

## 4. Results

Here we present our results. In Section 4.1, we detail our supporting result related to how different belief update models perform. Next, in Section 4.2, we present our main result about the trade-off between accuracy and risk in the Wisdom of the Crowd. Lastly, we present the supporting result regarding the effect of social learning during the high uncertainty period before the Brexit vote in Section 4.3.

### 4.1. Belief Update Models

Although the space of possible prior and likelihood distributions and posterior computation approaches is very large, we focus on using simple, interpretable, and theoretically motivated approaches from prior work [28]. We leave to future work the exploration of richer distributions and approaches to modeling belief update as it is beyond the scope of this study. We detail how model error and confidence intervals are evaluated in Supplementary Section A.3.3.

**Figure 3:**
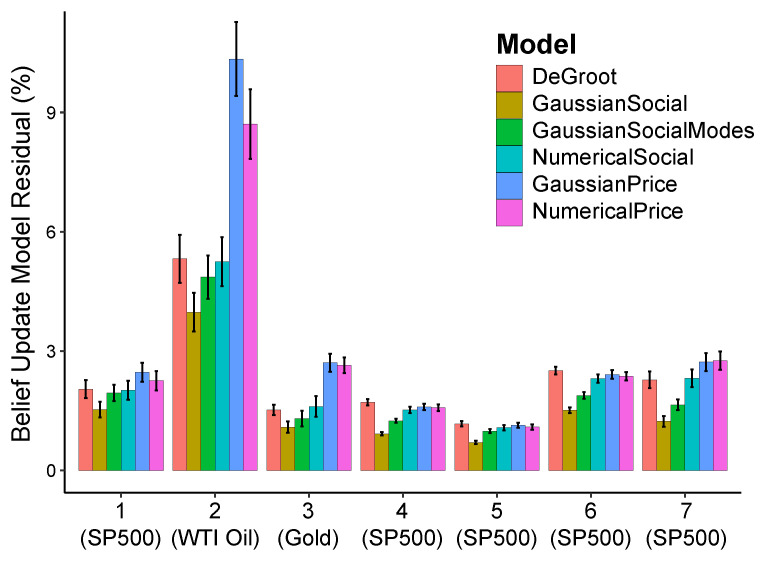
The y-axis shows the relative residual between *modeled* belief update and *actual* updated belief. Simple approximated models do better at modeling belief update than numerical models, and models using social histograms as likelihood perform better than models using the price history. Error bars represent 95% CI.

As can be seen in Figure 3, models that use social information as likelihood for modeling the belief update of participants (GaussianSocial,GaussianSocialModes, Numerical Social) outperform better than models that use the price history (GaussianPrice, Numerical Price). This suggests that our participants more likely use social information instead of the price history to update their belief, in line with previous work showing that participants often prefer using social information [71,72].

Specifically, GaussianSocial, our simple Gaussian model that assumes the data follows a single-mode Gaussian distribution, outperforms GaussianSocialModes, a model that identifies when the social histogram is non-unimodal (using the Hartigan’s dip test of unimodality [73]) and uses the largest mode as the mean of the distribution. This suggests that participants assume the data they learn from to be unimodal even when it is non-unimodal, in line with prior work [74,75] showing that this might be due to the fact that using multi-modal data is cognitively costly.

Additionally, GaussianSocial outperforms the more precise numerical model NumericalSocial which makes no parametric assumption on the data distributions and uses a Monte Carlo procedure to estimate the posterior distribution. This suggests that participants employ simple heuristics when learning from their peers, in line with the attribute substitution heuristic of human decision-making [30]. However, when participants are learning from the price history, the dominance of simpler models is not as clear because the performance of the simple GaussianPrice model is indistinguishable from that of the numerical model (NumericalPrice).

GaussianSocial also outperforms the popular DeGroot model commonly used as a benchmark in the literature [68], where an individual updates their belief as the weighted average belief of their peers. Here we set the weights (trust values) equal for all peers, as we have no data to estimate these weights, and therefore assume a uniform prior. It is interesting to note that GaussianSocial is equivalent to the DeGroot model when a participant’s weight on their own prior belief is equal to the total of the weights of all other participants. This agrees with previous work showing that participants put a disproportionately larger weight on their own prior belief [76,77].

Overall, the superiority of GaussianSocial in predicting belief update suggests that participants use a heuristic, unimodal, and simple belief update procedure when updating their beliefs, and that they predominantly update their predictions using social information instead of price history. It is important to note that approximate (non-Monte Carlo) models such as GaussianSocial and GaussianPrice are parameter-less models and did not require any parameter fitting, making their success in modeling belief update quite interesting.

### 4.2. Accuracy-Risk Trade-Off

Here, we present our main result about the trade-off between accuracy and risk in the Wisdom of the Crowd. Using a Pareto curve, we compare the improvement in prediction accuracy and risk (variance) of each subset $S_{\alpha_{s}}$ as defined by $\alpha_{s}$, a measure of the relative amount of social vs non-social learning.

**Figure 4:**
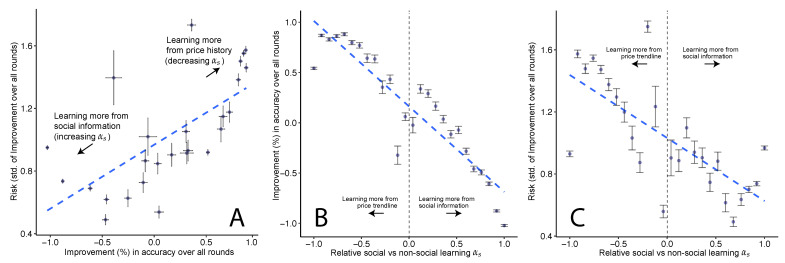
(**A**): In this Pareto curve, we plot the improvement of each subset vs. the risk (standard deviation) in improvement within this subset. We see a risk-return trade-off: predictions made with price history are more accurate, but with higher risk (standard deviation). Fitted line has $R^{2}$ of 0.49, and *p*-value < 0.001. Horizontal and vertical error bars represent 95% CI from 100 bootstraps. (**B**,**C**): Instead of plotting risk vs. improvement (as in (**A**), here we plot the same values of improvement ((**B**), $R^{2}$ = 0.82, *p*-value < 0.001) or risk ((**C**), $R^{2}$ = 0.50, *p*-value < 0.001) against the relative amount of social vs. non-social learning, $\alpha_{s}$, that generated these values of improvements or risk.

As shown in Figure 4, we observe that with improvements in accuracy of subsets comes increased risk, mediated by the relative amount of social vs. non-social learning $\alpha_{s}$, suggesting a trade-off between accuracy and risk. As formally described earlier in Section 3.3.3, improvement is a measure of the additional accuracy gained from a subset of predictions compared to when using all predictions by the crowd (the de-facto Wisdom of the Crowd) over all prediction rounds. Similarly, risk is a measure of the risk of this subset compared to when using all predictions over all rounds. From a system design perspective, we choose these measures of improvement and risk as they allow us to understand how choices over subsets of participants might affect performance, allowing us to calibrate the crowd as per the platform designer’s risk preferences.

Additionally, since we observe that variance of improvement (risk) decreases with increased social leaning, our result replicates prior findings that exposure to social information decreases the variance of the crowd [37]). Please note that the decrease in risk from social learning is not because participants are simply converging towards the crowd’s mean: as detailed in the previous Section 4.1, the social histogram participants are shown is quite often non-unimodal (tested using the Hartigan’s dip test of unimodality [73]), which means that participants are intentionally collapsing multiple distribution modes in the observed data.

Such a Pareto trade-off between risk and accuracy is common in financial forecasting [15,16] and statistical prediction [8,9,10,11], but has not been typically observed in the literature on the Wisdom of Crowds. This has strong implications for the design of crowd-sourced prediction platforms as described in the Discussion Section 5.1.

### 4.3. Performance under High Uncertainty

A supporting result of our work is from the investigation of the crowd’s performance during a period of high uncertainty using the data from the prediction round that happened during the Brexit vote (see Supplementary Section A.5 for details about this round).

Following the same procedure described in the Methods Section 3.3.3, we bin all α’s from the prediction sets and investigate the improvements of subsets of predictions compared to the whole crowd. The main difference here is that unlike in all previous results where we took care not to use the last week of data to calculate collective accuracy so that prediction was not too easy, we do so here as the high uncertainty only happened in the last week (as shown in Supplementary Figure S1). This last week of data that we use is a *disjoint subset* from the data we previously used.

**Figure 5:**
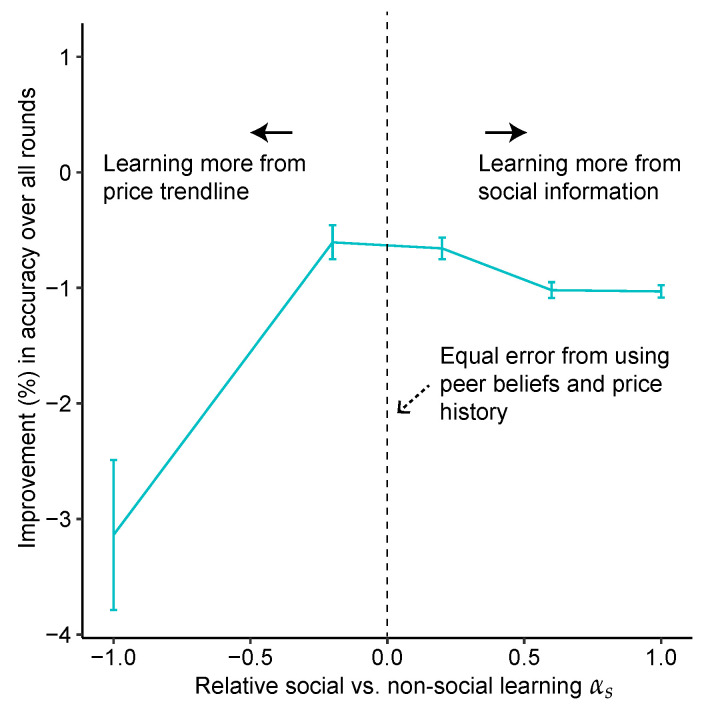
Improvement when selecting predictions based on how much more they were likely made using social information ($\alpha_{s}>0$) vs. price history ($\alpha_{s}<0$). 95% Confidence intervals obtained through 100 bootstraps.

As can be seen in Figure 5, as $\alpha_{s}$ decreases (i.e., we select predictions that were more likely updated using the price history instead of the social information, $\alpha_{s}<0$), improvement in accuracy of subsets compared to the Wisdom of the Crowd (all predictions) decays to a great extent.

Conversely, as subsets of predictions updated using the social histogram ($\alpha_{s}>0$) are selected, the improvement in their accuracy is stable.

Given that such high market uncertainty only occurred during one round, we do not have enough data to produce a Pareto curve over multiple rounds. Additionally, note that although a smaller number of predictions were made during the last week before Brexit (52 prediction sets compared to 284 during the open period of prediction used earlier), we have sufficient data to afford statistically significant results as shown by the 95% confidence intervals of our findings.

This supporting result suggests that during periods of high uncertainty, social learning leads to higher accuracy in contrast to the result in the previous section where the asset prices were more predictable. This result has implications for platform designers such as the potential of leveraging social learning as a valuable tool that minimizes catastrophic performance during high uncertainty prediction regimes.

## 5. Discussion

Our main result (the trade-off seen in Figure 4) supports our hypothesis that a Pareto frontier exists between risk and accuracy—similarly to what has been observed in statistical modeling [8,9,10] and financial [14,15,16] forecasting systems. This trade-off is mediated by the relative amount of social vs. non-social learning. Additionally, as supporting results, we observe that simple approximate models outperform more complicated Monte Carlo approaches in modeling the belief update process of participants. This suggests that participants use several heuristics, and that during periods of high uncertainty, social learning leads to higher accuracy.

Here, we discuss the implications of our results for platform designers in Section 5.1, describe the contributions of our work to the literature on heuristics in information processing and decision-making in Section 5.2. We end with a description the limitations of this work in Section 5.3.

### 5.1. Collective Intelligence System Design Implications

If we are to deploy crowd-sourced financial prediction and speculation systems at scale, it will be important to fully characterize the performance of these systems. This is especially given the growing importance of decentralized financial prediction and speculation including very recent events during which retail investors self-organized using social media and drove up asset and derivative prices [3,4]. However, crowd-sourced prediction systems and literature so far focus on measuring and optimizing for the accuracy of the predictions with little regard to the risk of these predictions even though measuring both accuracy and risk is standard in machine learning [8,9,10] and financial [14,15,16] forecasting applications. More generally, proper modeling and estimation of risk will support more sophisticated and versatile applications of crowd-sourced predictions such as hedging risks over portfolios of prediction tasks.

Additionally, beyond the passive monitoring and reporting of risk, a practical question for designers is how to *tune* the platform to reach a desired value of risk and accuracy. Our result that social learning can mediate the accuracy-risk trade-off provides a practical means to attain performance along this frontier. Specifically, our results suggest that social learning within a crowd-sourcing platform could be more purposefully leveraged to fit the task at hand. For example, platform designers could incentivize social learning between participants to have lower risk. This might be especially needed during highly uncertain times, as our results from the Brexit prediction (Figure 5) prediction showed. Past work has already showed that crowd-sourcing platforms can be incentivized to be more social [43,44].

Beyond platform design considerations, our results also add to the rich study of social learning and its impact on collective intelligence within the Wisdom of the Crowd domain [25,27,37,40,41] by adding the novel perspective that risk is an important dimension of the behavior of crowds to be measured.

More generally, our work brings together two disjoint studies by showing that it is possible to improve collective intelligence by modeling individual belief update. Our results therefore suggest a connection between the field of collective intelligence [78] (of which the Wisdom of the Crowd is one domain) and the field of computational cognitive science [79] (of which Bayesian models of cognition is an area). Until now, the latter literature has mostly focused on individual models of belief update such as through computational models of how people perform sampling [80], what their priors are [81], and how they perform inference [82], sometimes in social situations [83]. Yet, there is little work that looks at the impact of individual belief update on collective performance. On the other hand, there is limited collective intelligence literature regarding leveraging the modeling of individual belief update to improve group performance and past work has instead been focused on using personal characteristics such as resistance to social learning [27].

### 5.2. Information Processing and Decision-Making Heuristics

Our results also have implications for the literature on decision heuristics and biases [75,84]. Through the modeling of belief update, we observe that our subjects exhibit the attribute substitution heuristic of human decision-making [30]. This information processing heuristic describes when people attempt to solve a complicated problem by approximating it with a simpler, less accurate model. We observe this heuristic as our participants’ updated beliefs are better modeled by the GaussianSocial model (which assumes the data to be unimodal) than by the multi-modal belief update model GaussianSocialModes. This indicates that our participants assume the data to be unimodal even when it is not, in line with previous studies that have shown that people wrongly assume data to be unimodal [74,85,86]. This is hypothesized to be because updating belief using multi-modal data is cognitively costly [87]. Additional evidence of this substitution heuristic is from the fact that simpler, approximate models better predict the updated beliefs of participants than the more complicated Monte Carlo numerical models.

Another decision heuristic that we observe is that participants prefer to use social information rather than the underlying price history of an asset to update their belief as models which use social information (GaussianSocial,GaussianSocialModes, and NumericalSocial) outperform models that use price history (GaussianPrice and NumericalPrice) as shown in Figure 3. This is surprising given that our participants were mid-career finance professionals with strong financial experience who should know that price information is generally better to predict future prices [88,89]). However, such behavior was observed in prior work where even experts performing a familiar task demonstrate sub-optimal decision heuristics [90,91], and often over-rely on social information [71,72].

Generally, such information processing and decision-making heuristics have been seen as irrational and sub-optimal. Our results suggest that within the full specification of both accuracy and risk, perhaps participants are preferentially aiming for lower risk instead of higher accuracy. This preference for social information especially pays off during the high uncertainty period before the Brexit vote. Our results support growing evidence that heuristics and biases are not merely *defects* of human decision-making, but that perhaps they optimize for richer objectives or are optimized for more time- or data-constrained decision-making [92,93,94,95,96,97,98]. For example, when individual decision-making is viewed within the lens of more realistic requirements such as limited time [99,100] or attention [101], heuristics and biases have been shown to act as helpful priors that facilitate fast and risk-averse decision-making [102,103].

### 5.3. Limitations and Future Work

We made several simplifying assumptions in this work that open up rich avenues for future work. First, we used simple, interpretable, and theoretically motivated belief update modeling approaches from prior work [28] and leave to future work the exploration of richer models, distributions and posterior computations to investigate belief update. One important set of models to investigate is the use of log-normal distributions for the likelihood instead of the normal distributions used in this work due to the established tendency of people to guess quantities log-normally [37,104,105]. Similarly, people have been shown to incorporate information asymmetrically based on where their predictions lie in relation to the information they are exposed to [106]. Overall, although we used Gaussian models here, an interesting direction of future work would be to build on the rich existing literature on how people incorporate information [84,107,108]. We also restricted each round to have a static population of participants whose predictions were shared using a specific visualization. An interesting direction for future work would be to embed participants in social networks given the importance and popularity of recent work on the effect of communication topologies [25,41,42,109] on group performance. Similarly, it would be interesting to investigate if different avenues for communication (e.g., discussions on forums [110]) exhibit a similar accuracy-risk trade-off.

Although this work demonstrates that our simple estimation technique can be used to tune crowd-predictions for desired levels of accuracy and risk, there are potential causal issues that could be improved in our experimental design and data analysis. One such issue is that there are two experimental and two analysis factors being investigated simultaneously here. These are the two different treatments in the form of sources of information (peer beliefs for the social histogram and price trajectory from the past price history) and the two different approaches through which each of these sources of information are being processed (simple binning of peer beliefs into a histogram, and transformation of the price history into a ‘rates histogram’). It can be argued that these two experimental treatments and two approaches constitute four possible approaches of how to deploy and analyze an experiment, and we have only compared two of these four approaches. From a scholarly perspective, we believe that our paper still makes a contribution because the goal of this work was to show that a trade-off exists and is mediated by social learning. We achieve this goal even though we only compare two approaches. Another causal concern is that the two experimental treatments might interact in non-trivial ways. For example, when visualized as a causal graph, there might be causally confounding paths between the treatments.

Several research designs and estimations techniques exist to remedy these causal limitations. One approach would be to use an A/B test [64] framework although it would require exposing people to different information separately. Doing so would be against our goal to investigate how people update their belief in real-life situations where users are exposed to both social information and price history. However, experiments where different types of information are shown separately could still be used to understand the effect of different information exposures on accuracy and risk, and used in deployment. Similarly different amounts of information exposure could be attempted using a multi-factorial A/B test [111,112]. We leave the exploration of these more sophisticated designs to future work. Other de-confounding approaches could involve assuming a causal graph [113] that is believed to capture how people update information and to use causal tools such as d-separation to estimate the effect of different information exposure. Another approach would be to use a potential outcomes framework [114] to estimate these treatments. These are promising directions of research which could be investigated using our data that we leave to future work. From a platform design perspective, even though these confounding issues remain, our estimation technique could be readily applied to crowd-sourced systems where price histories and peer beliefs are being shown.

## Data Availability

Not applicable.

## Supplementary Materials

The following are available online at https://www.mdpi.com/article/10.3390/e23070801/s1. References [115,116,117,118,119,120] are cited in the supplementary materials.
