# Supplementary material for: Accuracy-Risk Trade-Off Due to Social Learning in Crowd-Sourced Financial Predictions
Source: Entropy (Basel). 2021 Jun 24;23(7):801. doi: 10.3390/e23070801 (PMC8307866; doi:10.3390/e23070801)
Supplement: Supplementary file 1 [file entropy-23-00801-s001.zip › entropy-1253219-supplementary.pdf]

entropy

MDPI

Article

Accuracy-Risk Trade-Off due to Social Learning in Crowd-Sourced Financial Predictions

Dhaval Adjodah [1,*], Yan Leng [2], Shi Kai Chong [1], P. M. Krafft [3], Esteban Moro [4], Alex Pentland [1,*]

[1] Media Lab, Massachusetts Institute of Technology, Cambridge, MA 02139; cshikai@mit.edu
[2] McCombs School of Business, The University of Texas at Austin, Austin, TX 78712, USA; yleng@mit.edu
[3] Oxford Internet Institute, University of Oxford, Oxford OX1 2JD, UK; pkrafft@mit.edu
[4] Departamento de Matemáticas & GISC, Universidad Carlos III de Madrid, 28911 Leganes, Spain; emoro@mit.edu
* Correspondence: dval@mit.edu (D.A.); pentland@mit.edu (A.P.)

check for updates

Citation: Adjodah, D.; Leng, Y.; Chong, S.K.; Krafft, P. M.; Moro, E,; Pentland, A. Accuracy-Risk Trade-Off due to Social Learning in Crowd-Sourced Financial Predictions. *Entropy* **2021**, *23*, 801. https://doi.org/10.3390/e23070801

Academic Editor: Stanisław Drożdż

Received: 25 May 2021
Accepted: 17 June 2021
Published: 24 June 2021

Publisher's Note: MDPI stays neutral with regard to jurisdictional claims in published maps and institutional affiliations.

Copyright: © 2021 by the authors. Licensee MDPI, Basel, Switzerland. This article is an open access article distributed under the terms and conditions of the Creative Commons Attribution (CC BY) license (https://creativecommons.org/licenses/by/4.0/).

A. Supplementary Material

A.1. External Validity of Data Collected

In Table S1, we summarize information about the asset prices predicted and the various measures of accuracy. Overall, our participants are collectively accurate — in agreement with past Wisdom of the Crowd studies [23,24] — indicating that their predictions are being thoughtfully elicited. This is evidenced by the fact that

- The crowd's mean prediction error is much less than the overall price change of the assets for the 3-week prediction period.
- The crowd is generally doing more than just linear extrapolation as their error is smaller than than from a linear extrapolation model (using a first order price momentum prediction).
- The crowd's collective prediction error over each round tracks (and sometimes outperforms) the futures of each asset being predicted (we calculate the futures error as the difference between the futures price and the asset price). Because futures prices are commonly used a measure of the global market's prediction of the price asset [115,116], the fact that the crowd's performance is on-par with the futures prices indicates that our dataset is externally valid.

Note that the higher relative errors in round 2 are an artifact of the fact that a few dollars' error on the lower price (about $45 per share) of WTI Oil seems like a higher error compared to same absolute error on the higher prices of the other assets ($1300 per gold share and $2100 per S&P 500 share).

Table S1: Summary of data collected. Our crowd is accurate, and sometimes even outperforms the futures underlying the asset.

	Prediction Round						
	**1**	**2**	**3**	**4**	**5**	**6**	**7**
**Asset**	**S&P 500**	**WTI Oil**	**Gold**	**S&P 500**	**S&P 500**	**S&P 500**	**S&P 500**
**Grounth Truth**	2037.41	45.95	1335.80	2153.74	2126.41	2191.95	2262.53
**Num. Prediction Sets**	284	207	134	1174	925	1441	469
**Price Change (%)**	4.01	11.03	3.63	1.77	1.75	2.24	3.56
**Linear Extrapolation Error (%)**	6.66	16.4	1.26	1.62	2.75	0.75	3.10
**Crowd Mean Error (%)**	2.22	4.95	0.46	0.84	0.58	3.20	2.40
**Futures Mean Error (%)**	2.03	3.05	0.94	0.38	0.40	0.48	1.50

Additionally, whenever we predict a final closing price, we only use participant prediction data up to the week before the day of prediction (i.e., we don't use any data during the last week of the round) so that our predictions are not too easy. We use each asset's futures price as a proxy for the prediction of the entire market to test the external validity of our crowd's predictions. To do so, we chose the start and end dates of each round so that the expiry dates of the asset's underlying *futures* would not affect the price of

both the asset and its futures. Financial data (asset and futures prices) is obtained through Barchart.com's API.

A.2. Momentum Transformation of Price History

When using price history B_T, the time-series of prices needs to first be transformed into a cognitively-interpretable likelihood distribution [65]. A number of methodologies and approaches exist, and here we use a simple, interpretable, and theoretically-motivated approach from prior work [66,67] where it has been shown that people process time-series as a distribution of changes as opposed to a distribution of the quantity itself. In our case, this means that we cannot just use the prices from the price history as a histogram, and should instead create a histogram of daily changes (slopes) in prices. Therefore, for each day's price B_t, a daily rate, r_t, of asset price change is calculated for each day t during the 6-month interval that a participant is shown, $r_t = \frac{B_t - B_{t-1}}{B_t}$. Then these rates are used to create a histogram (similar to the histogram of social information) and this rate histogram is utilized for both the simple Gaussian models and the numerical models.

In the simple Gaussian models the mean of this histogram (which is the mean rate $\bar{r}_t$ over the 6 month period) is multiplied by the number of days between the pre-exposure prediction and the final day of the prediction round (for when the asset's price is being predicted) to obtain the post-exposure prediction, $B^{pred}_{post} = B_{pre} + B_{pre}.\bar{r}_t.n_{days}$. The same calculation is done for the numerical model, but for each bin in the rates histogram.

A.3. Modeling Belief Update

Here we formally describe the derivation of the simple Gaussian models and the numerical models and how the posterior is estimated.

A.3.1. Approximate Gaussian Approach:

We describe `GaussianSocial` here. `GaussianPrice` follows the same derivation, substituting the social histogram B_H with the price history B_T.

Our notation here follows that of [117]. We assume that people's estimate of the future price before information exposure, B_{pre}, is being sampled from an internal prior distribution [80], and that the sample we obtain is indicative of the mean of the prior distribution following the results of [118].

We suppose that people think each asset has a true value, V^*, which people are trying to estimate to predict the future asset value, V (the ground truth); that prior beliefs about V^* follow a Normal (Gaussian) prior distribution, $V^* \sim Normal(\mu_{prior}, \sigma_{prior})$; and that evidence about V^* can be understood as being generated from a Normal distribution, $Normal(V^*, \sigma_{data})$. In this case the posterior beliefs people have follows a simple form. Letting information content be defined as the inverse of the Normal distribution's variance $I = \frac{1}{\sigma}$, we have that

$$\mu_{posterior} = \frac{\mu_{prior} \cdot I_{prior} + \mu_{data} \cdot I_{data}}{I_{prior} + I_{data}}. \tag{1}$$

Additionally, the social histogram is treated as representing the information content of data about V^*, then we have:

$$\mu_{posterior} = \frac{B_{pre} \cdot I_{prior} + \overline{B_H} \cdot I_{data}}{I_{prior} + I_{data}}. \tag{2}$$

The `GaussianSocial` rule therefore can be viewed as reflecting an assumption of a Normal distribution as a mental model, and assuming private information and social information have the same information content ($I_{prior} = I_{data}$), which gives:

$$\mu_{posterior} = \frac{B_{pre} + \overline{B_H}}{2}. \tag{3}$$

Although simple, this belief update approach has been shown to faithfully model people's belief update in a variety of domains from predicting movie grossing revenue to pharaoh reign lengths. [28,29]

A.3.2. Numerical Approach:

Here, we can use a numerical approach by binning the likelihood distributions to estimate the posterior distribution using Monte Carlo methods. Because we do not have access to the distribution of the prior belief of each participant (as we only have an individual point estimate for each prediction set), we still have to approximate the prior. We model the prior to be Gaussian, with the mean set as the pre-exposure prediction of a participant, B_{pre}, and the standard deviation set as the standard deviation of the social histogram B_H or the standard deviation of the price history B_T, depending on which likelihood distribution we are modeling with.

We estimate the posterior distribution $P_{posterior}(b)$ of a participant's post-exposure prediction b in the following way: let b_j be a unique value in $\mathbf{B_H}$, and $P_{B_H}(b_h)$ be the probability density of b_h in $\mathbf{B_H}$. Let $P_{prior}(b)$ be the density of b in the parametrized prior distribution. The posterior distribution for the numerical model is defined as $P_{posterior}(b) = \frac{P_{B_H}(b) \times P_{prior}(b)}{\sum_{b_j \in B_H} P_{B_H}(b_j) \times P_{prior}(b_j)}$ when using the social information B_H. The distributions is simple enough that sampling was not needed. After computing this posterior distribution, we use the mean of the distribution as the *modeled* updated belief of a participant.

A.3.3. Evaluating Model Error

For all models, we compute the residual error between the model's prediction of the posterior ($\mu_{\sim P_{posterior}(V)}$) and the actual post-exposure prediction (B_{post}) as: $(\mu_{\sim P_{posterior}(V)} - B_{post})/B_{post}$. For the approximate approach, $\mu_{\sim P_{posterior}(V)}$ is simply the mean of the normal distribution representing the posterior, while in the numerical approach, the mean is estimated through averaging over all bins of the empirical distribution (the distribution is small enough that sampling was not needed).

For all models, the 95% confidence intervals are calculated as follows: we assume the data follows Student's t-distribution since the variance of the true distribution is unknown and, therefore, we estimate it from the sample data. Let s_e be the estimated standard error of the sample mean and t_e be the t-value for the 95% confidence interval desired, which can be computed via inverse t-distribution. The lower and upper limits for the 95% confidence interval are $[\mu_e - t_e s_e, \mu_e + t_e s_e]$, where μ_e is the estimated sample mean.

Table S2: Values of the residual for each round for all models. Numbers in parentheses show the 95% confidence interval.

MODEL	ROUND						
	1 (S&P 500)	2 WTI Oil	3 Gold	7 (S&P 500)	8 (S&P 500)	9 (S&P 500)	12 (S&P 500)
GaussianSocial	1.53 (0.19)	3.97 (0.48)	1.08 (0.13)	0.92 (0.04)	0.70 (0.04)	1.51 (0.07)	1.23 (0.13)
GaussianSocialModes	1.94 (0.20)	4.85 (0.54)	1.30 (0.19)	1.24 (0.05)	0.98 (0.04)	1.88 (0.08)	1.64 (0.13)
NumericalSocial	2.01 (0.23)	5.24 (0.61)	1.60 (0.25)	1.52 (0.08)	1.07 (0.06)	2.31 (0.10)	2.31 (0.22)
NumericalPrice	2.25 (0.23)	8.70 (0.87)	2.64 (0.19)	1.57 (0.08)	1.09 (0.06)	2.36 (0.10)	2.75 (0.23)
GaussianPrice	2.46 (0.24)	10.3 (0.92)	2.70 (0.22)	1.59 (0.07)	1.13 (0.06)	2.41 (0.10)	2.72 (0.22)
DeGroot	2.04 (0.22)	5.32 (0.60)	1.52 (0.13)	1.71 (0.07)	1.17 (0.06)	2.51 (0.09)	2.27 (0.21)

A.3.4. Boostrapping

Our reported value of improvement (the one in Fig. 4) is over 100 random bootstraps with replacement.

As defined in section 3.3.3 in the main text, the error of a subset S_{α_s} is

$$e_{i,S_{\alpha_s}} = \frac{|\sum_{j \in S_{\alpha_s}} [B_{post,j}] - V_i|}{V_i} \tag{4}$$

and the error for all predictions, the Wisdom of the Crowd is:

$$e_{i,S_{all}} = \frac{|\sum_{j \in S_{all}}[B_{post,j}] - V_i|}{V_i} \tag{5}$$

Which means that the improvement of a subset, within a bootstrap b for a particular round i is

$$I_{b,i}^{S_{\alpha_s}} = |e_{i,S_{\alpha_s}} - e_{i,S_{all}}| \tag{6}$$

In Fig 4, we report the average improvement in accuracy over rounds i and over bootstraps b, $\sum_b[\sum_i[I_{b,i}^{S_{\alpha_s}}]]$. Similarly, the risk of this subset S_{α_s} over rounds i is $\sqrt{\sum_i[(I_{b,i}^{S_{\alpha_s}} - \sum_i[I_{b,i}^{S_{\alpha_s}}])^2]}$. We use standard deviation instead of variance as it is the more popular measure of risk in practice [15]. For both the average improvement and the risk, we obtain the 95% confidence intervals over the bootstraps.

A.4. Table of Subset Improvement and Risk

Table S3: Improvements achieved by subsetting predictions via α_s for all rounds. 95% confidence intervals are calculated through 100 bootstraps.

α_s	**Improvement**	**Improvement CI**	**Risk**	**Risk CI**
-1.00	0.526	0.015	0.914	0.019
-0.92	0.866	0.013	1.584	0.021
-0.84	0.839	0.018	1.463	0.029
-0.76	0.882	0.014	1.552	0.025
-0.68	0.880	0.017	1.467	0.028
-0.60	0.798	0.019	1.408	0.038
-0.52	0.734	0.031	1.254	0.060
-0.44	0.682	0.038	1.186	0.069
-0.36	0.607	0.040	1.060	0.081
-0.28	0.344	0.067	0.941	0.073
-0.20	0.435	0.041	1.744	0.039
-0.12	-0.382	0.079	1.344	0.160
-0.04	0.032	0.044	0.498	0.035
0.04	-0.161	0.093	0.952	0.138
0.12	0.292	0.052	0.882	0.068
0.20	0.274	0.037	1.043	0.067
0.28	0.145	0.041	0.923	0.073
0.36	0.044	0.036	0.891	0.059
0.44	-0.073	0.039	0.793	0.060
0.52	-0.095	0.035	0.858	0.073
0.60	-0.238	0.034	0.605	0.051
0.68	-0.432	0.031	0.493	0.029
0.76	-0.460	0.023	0.606	0.032
0.84	-0.620	0.018	0.692	0.017
0.92	-0.885	0.011	0.742	0.014
1.00	-1.021	0.009	0.963	0.012

Table S4: Improvements achieved by subsetting predictions via α_s only for predictions the week before Brexit. Confidence intervals are calculated through 100 bootstraps.

α_s	**Improvement (%)**	**CI**
-1.0	-3.14	0.65
-0.2	-0.61	0.15
0.2	-0.66	0.09
0.6	-1.02	0.07
1.0	-1.03	0.05

A.5. Brexit Data

We deployed one of our experiments right before the Brexit vote during which there was a lot of market uncertainty [31]: the prediction round starting on June 1st 2016 ended on June 24th 2016, the day of the Brexit vote, and participants were predicting the price of the S&P 500, an asset sensitive to global events [119,120].

We collected 284 prediction sets during the first 2 weeks of the round, and 52 sets in the last week during which the global financial market first overestimated then underestimated the final price of the S&P 500 asset leading to a 3.7% crash, as shown in the candlestick plot in Fig. S1.

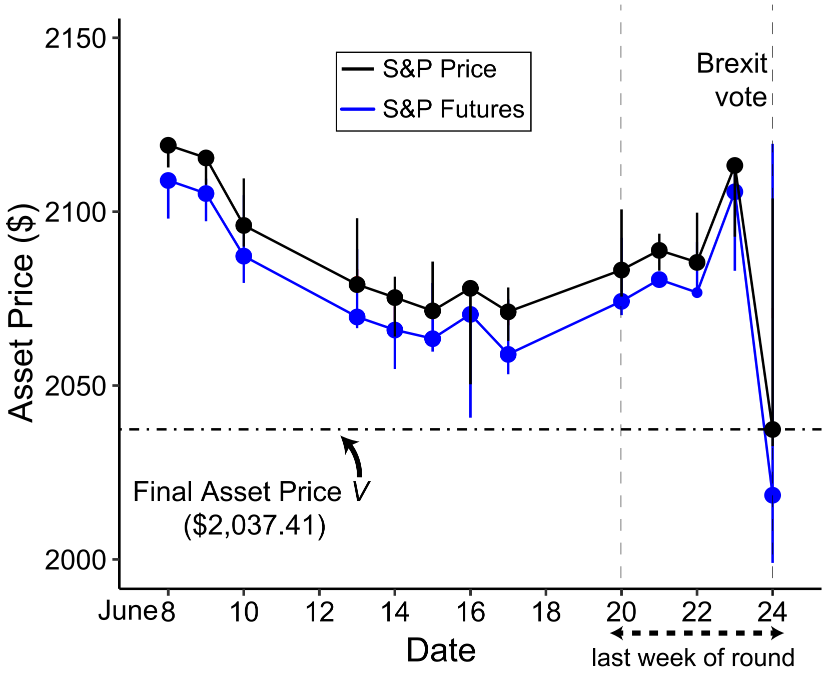

Figure S1. The close, low, and high price of the asset and its underlying futures are shown as candlestick plots. The asset and futures overestimated the price and then crashed during the last week.

References

1. Diallo, N.; Shi, W.; Xu, L.; Gao, Z.; Chen, L.; Lu, Y.; Shah, N.; Carranco, L.; Le, T.C.; Surez, A.B.; et al. eGov-DAO: A better government using blockchain based decentralized autonomous organization. In Proceedings of the 2018 International Conference on eDemocracy & eGovernment (ICEDEG), Ambato, Ecuador, 4–6 April 2018; pp. 166–171.
2. Lang, M.; Bharadwaj, N.; Di Benedetto, C.A. How crowdsourcing improves prediction of market-oriented outcomes. *J. Bus. Res.* **2016**, *69*, 4168–4176.
3. Lawrence, K. *Memes, Reddit, and Robinhood: Analyzing the GameStop Saga*; 2021. Available online: http://sk.sagepub.com/cases/memes-reddit-and-robinhood-analyzing-the-gamestop-saga (accessed on 10 May 2021)
4. Hu, D.; Jones, C.M.; Zhang, V.; Zhang, X. The Rise of Reddit: How Social Media Affects Retail Investors and Short-Sellers' Roles in Price Discovery. 2021. Available online : https://papers.ssrn.com/sol3/papers.cfm?abstract_id=3807655 (accessed on 10 May 2021)
5. Lazer, D.; Friedman, A. The network structure of exploration and exploitation. *Adm. Sci. Q.* **2007**, *52*, 667–694.
6. Olorunda, O.; Engelbrecht, A.P. Measuring exploration/exploitation in particle swarms using swarm diversity. In Proceedings of the 2008 IEEE Congress on Evolutionary Computation (IEEE World Congress on computational intelligence), Hong Kong, China, 1–6 June 2008; pp. 1128–1134.
7. Kennedy, J.; Mendes, R. Population structure and particle swarm performance. In Proceedings of the 2002 Congress on Evolutionary Computation, CEC'02 (Cat. No. 02TH8600), Honolulu, HI, USA, 12–17 May 2002; Volume 2, pp. 1671–1676.
8. James, G.; Witten, D.; Hastie, T.; Tibshirani, R. *An Introduction to Statistical Learning*; Springer: Berlin/Heidelberg, Germany, 2013; Volume 112.
9. Domingos, P. A unified bias-variance decomposition. In Proceedings of 17th International Conference on Machine Learning, Stanford, CA, USA, 29 June – July 2 2000; pp. 231–238.

10. Geman, S.; Bienenstock, E.; Doursat, R. Neural networks and the bias/variance dilemma. *Neural Comput.* **1992**, *4*, 1–58.
11. Gagliardi, F. Instance-based classifiers applied to medical databases: diagnosis and knowledge extraction. *Artif. Intell. Med.* **2011**, *52*, 123–139.
12. Markowitz, H. Portfolio selection. *J. Financ.* **1952**, *7*, 77–91.
13. Gammerman, A.; Vovk, V. Hedging predictions in machine learning. *Comput. J.* **2007**, *50*, 151–163.
14. Joyce, J.M.; Vogel, R.C. The uncertainty in risk: Is variance unambiguous? *J. Financ.* **1970**, *25*, 127–134.
15. Modigliani, F.; Leah, M. Risk-adjusted performance. *J. Portf. Manag.* **1997**, *23*, 45.
16. Ghysels, E.; Santa-Clara, P.; Valkanov, R. There is a risk-return trade-off after all. *J. Financ. Econ.* **2005**, *76*, 509–548.
17. Chavez-Demoulin, V.; Embrechts, P.; Nešlehová, J. Quantitative models for operational risk: extremes, dependence and aggregation. *J. Bank. Financ.* **2006**, *30*, 2635–2658.
18. Asmussen, S.; Kroese, D.P. Improved algorithms for rare event simulation with heavy tails. *Adv. Appl. Probab.* **2006**, *38*, 545–558.
19. Shevchenko, P.V.; Wuthrich, M.V. The structural modelling of operational risk via Bayesian inference: Combining loss data with expert opinions. *J. Oper. Risk* **2006**, *1*, 3–26.
20. Chapelle, A.; Crama, Y.; Hübner, G.; Peters, J.P. Practical methods for measuring and managing operational risk in the financial sector: A clinical study. *J. Bank. Financ.* **2008**, *32*, 1049–1061.
21. Cruz, M.G. *Modeling, Measuring and Hedging Operational Risk*; Wiley: New York, NY, USA, 2002; Volume 346.
22. Galton, F. Vox populi (The wisdom of crowds). *Nature* **1907**, *75*, 450–451.
23. Golub, B.; Jackson, M.O. Naive learning in social networks and the wisdom of crowds. *Am. Econ. J. Microecon.* **2010**, *2*, 112–149.
24. Nofer, M.; Hinz, O. Are crowds on the internet wiser than experts? The case of a stock prediction community. *J. Bus. Econ.* **2014**, *84*, 303–338.
25. Becker, J.; Brackbill, D.; Centola, D. Network dynamics of social influence in the wisdom of crowds. *Proc. Natl. Acad. Sci. USA* **2017**, *114*, 201615978; doi:10.1073/pnas.1615978114.
26. Turner, B.M.; Steyvers, M.; Merkle, E.C.; Budescu, D.V.; Wallsten, T.S. Forecast aggregation via recalibration. *Mach. Learn.* **2014**, *95*, 261–289.
27. Madirolas, G.; de Polavieja, G.G. Improving collective estimations using resistance to social influence. *PLoS Comput. Biol.* **2015**, *11*, e1004594.
28. Griffiths, T.L.; Tenenbaum, J.B. Optimal predictions in everyday cognition. *Psychol. Sci.* **2006**, *17*, 767–773, doi:10.1111/j.1467-9280.2006.01780.x.
29. Griffiths, T.L.; Kemp, C.; Tenenbaum, J.B. Bayesian models of cognition. In *The Cambridge Handbook of Computational Psychology*; Sun, R., Ed.; Cambridge University Press: Cambridge, UK , 2008; pp. 1–49.
30. Kahneman, D.; Frederick, S. *Representativeness Revisited: Attribute Substitution in Intuitive Judgment*; doi:10.1017/CBO9780511808098.004.
31. Oehler, A.; Horn, M.; Wendt, S. Brexit: Short-term stock price effects and the impact of firm-level internationalization. *Financ. Res. Lett.* **2017**, *22*, 175–181.
32. Kennedy, J. Swarm intelligence. In *Handbook of Nature-Inspired and Innovative Computing*; Springer: Berlin/Heidelberg, Germany, 2006; pp. 187–219.
33. Eberhart, R.C.; Shi, Y.; Kennedy, J. *Swarm Intelligence*; Elsevier: Amsterdam, The Netherlands, 2001.
34. Bonabeau, E.; Marco, D.D.R.D.F.; Dorigo, M.; Théraulaz, G.; Theraulaz, G.; others. *Swarm Intelligence: From Natural to Artificial Systems*; Number 1; Oxford University Press: Oxford, UK, 1999.
35. Woolley, A.W.; Chabris, C.F.; Pentland, A.; Hashmi, N.; Malone, T.W. Evidence for a collective intelligence factor in the performance of human groups. *Science* **2010**, *330*, 686–688.
36. Malone, T.W.; Laubacher, R.; Dellarocas, C. The collective intelligence genome. *MIT Sloan Manag. Rev.* **2010**, *51*, 21.
37. Lorenz, J.; Rauhut, H.; Schweitzer, F.; Helbing, D. How social influence can undermine the wisdom of crowd effect. *Proc. Natl. Acad. Sci. USA* **2011**, *108*, 9020–9025; doi:10.1073/pnas.1008636108.
38. Muchnik, L.; Aral, S.; Taylor, S.J. Social influence bias: A randomized experiment. *Science* **2013**, *341*, 647–651.
39. Salganik, M.J.; Dodds, P.S.; Watts, D.J. Experimental study of inequality and unpredictability in an artificial cultural market. *Science* **2006**, *311*, 854–856.
40. Moussaïd, M.; Kämmer, J.E.; Analytis, P.P.; Neth, H. Social influence and the collective dynamics of opinion formation. *PloS one* **2013**, *8*, e78433, doi:10.1371/journal.pone.0078433.
41. Almaatouq, A.; Noriega-Campero, A.; Alotaibi, A.; Krafft, P.; Moussaid, M.; Pentland, A. Adaptive social networks promote the wisdom of crowds. *Proc. Natl. Acad. Sci. USA* **2020**, *117*, 11379–11386.
42. Adjodah, D.; Calacci, D.; Dubey, A.; Goyal, A.; Krafft, P.; Moro, E.; Pentland, A. Leveraging Communication Topologies Between Learning Agents in Deep Reinforcement Learning. In Proceedings of the 19th International Conference on Autonomous Agents and MultiAgent Systems, Auckland, New Zealand, 9–13 May 2020.
43. Lim, S.L.; Quercia, D.; Finkelstein, A. StakeSource: harnessing the power of crowdsourcing and social networks in stakeholder analysis. In Proceedings of the 2010 ACM/IEEE 32nd International Conference on Software Engineering, Cape Town, South Africa, 2–8 May 2010; Volume 2, pp. 239–242.
44. Chen, P.Y.; Cheng, S.M.; Ting, P.S.; Lien, C.W.; Chu, F.J. When crowdsourcing meets mobile sensing: A social network perspective. *IEEE Commun. Mag.* **2015**, *53*, 157–163.

45. Lerman, K.; Ghosh, R. Information contagion: An empirical study of the spread of news on Digg and Twitter social networks. In Proceedings of the Fourth International AAAI Conference on Weblogs and Social Media (ICWSM), Washington, DC, USA, 23–26 May 2010.
46. Lerman, K.; Hogg, T. Using a model of social dynamics to predict popularity of news. In Proceedings of the 19th International Conference on World Wide Web (WWW), Raleigh, NC, USA, 26–30 April 2010; pp. 621–630.
47. Stoddard, G. Popularity dynamics and intrinsic quality in reddit and hacker news. In Proceedings of the Ninth International AAAI Conference on Web and Social Media (ICWSM), Oxford, UK, 26–29 May 2015.
48. Celis, L.E.; Krafft, P.M.; Kobe, N. Sequential voting promotes collective discovery in social recommendation systems. In Proceedings of the Tenth International AAAI Conference on Web and Social Media, Cologne, Germany, 17–20 May 2016.
49. Karger, D.R.; Oh, S.; Shah, D. Budget-optimal task allocation for reliable crowdsourcing systems. *Oper. Res.* **2014**, *62*, 1–24.
50. Holt, C.A.; Laury, S.K. Risk aversion and incentive effects. *Am. Econ. Rev.* **2002**, *92*, 1644–1655.
51. Kahneman, D.; Tversky, A. Prospect theory: An analysis of decision under risk. In *Handbook of the Fundamentals of Financial Decision Making: Part I*; World Scientific: Singapore, 2013; pp. 99–127.
52. Hintze, A.; Olson, R.S.; Adami, C.; Hertwig, R. Risk sensitivity as an evolutionary adaptation. *Sci. Rep.* **2015**, *5*, 8242.
53. Zhang, R.; Brennan, T.J.; Lo, A.W. The origin of risk aversion. *Proc. Natl. Acad. Sci. USA* **2014**, *111*, 17777–17782.
54. Binswanger, H.P.; Sillers, D.A. Risk aversion and credit constraints in farmers' decision-making: A reinterpretation. *J. Dev. Stud.* **1983**, *20*, 5–21.
55. Armstrong, J.S.; Green, K.C.; Graefe, A. Golden rule of forecasting: Be conservative. *J. Bus. Res.* **2015**, *68*, 1717–1731.
56. Passino, K.M.; Seeley, T.D. Modeling and analysis of nest-site selection by honeybee swarms: the speed and accuracy trade-off. *Behav. Ecol. Sociobiol.* **2006**, *59*, 427–442.
57. Valentini, G.; Hamann, H.; Dorigo, M. Efficient decision-making in a self-organizing robot swarm: On the speed versus accuracy trade-off. In Proceedings of the 2015 International Conference on Autonomous Agents and Multiagent Systems, Istanbul, Turkey, 4–8 May 2015; pp. 1305–1314.
58. Krause, J.; Ruxton, G.D.; Krause, S. Swarm intelligence in animals and humans. *Trends Ecol. Evol.* **2010**, *25*, 28–34.
59. Wolf, M.; Kurvers, R.H.; Ward, A.J.; Krause, S.; Krause, J. Accurate decisions in an uncertain world: Collective cognition increases true positives while decreasing false positives. *Proc. R. Soc. B Biol. Sci.* **2013**, *280*, 20122777.
60. Ward, A.J.; Herbert-Read, J.E.; Sumpter, D.J.; Krause, J. Fast and accurate decisions through collective vigilance in fish shoals. *Proc. Natl. Acad. Sci. USA* **2011**, *108*, 2312–2315.
61. Dumas, B.; Fleming, J.; Whaley, R.E. Implied volatility functions: Empirical tests. *J. Financ.* **1998**, *53*, 2059–2106.
62. Campbell, J.Y.; Shiller, R.J. Stock prices, earnings, and expected dividends. *J. Financ.* **1988**, *43*, 661–676.
63. Fama, E.F. Random walks in stock market prices. *Financ. Anal. J.* **1995**, *51*, 75–80.
64. Dixon, E.; Enos, E.; Brodmerkle, S. A/b Testing of a Webpage. US Patent 7,975,000, 2013. Available online: https://patents.google.com/patent/US20060162071A1/en (accessed on 10 May 2021)
65. Maniadakis, M.; Trahanias, P. Time models and cognitive processes: A review. *Front. Neurorobotics* **2014**, *8*, 7.
66. Park, C.H.; Irwin, S.H. What do we know about the profitability of technical analysis? *J. Econ. Surv.* **2007**, *21*, 786–826.
67. Neftci, S.N. Naive trading rules in financial markets and wiener-kolmogorov prediction theory: A study of "technical analysis". *J. Bus.* **1991**, pp. 549–571.
68. DeGroot, M.H. Reaching a consensus. *J. Am. Stat. Assoc.* **1974**, *69*, 118–121.
69. Kerckhove, C.V.; Martin, S.; Gend, P.; Rentfrow, P.J.; Hendrickx, J.M.; Blondel, V.D. Modelling influence and opinion evolution in online collective behaviour. *PLoS ONE* **2016**, *11*, e0157685.
70. Soll, J.B.; Larrick, R.P. Strategies for revising judgment: How (and how well) people use others' opinions. *J. Exp. Psychol. Learn. Mem. Cogn.* **2009**, *35*, 780.
71. Foster, F.D.; Viswanathan, S. Strategic trading when agents forecast the forecasts of others. *J. Financ.* **1996**, *51*, 1437–1478.
72. Posada, M.; Hernandez, C.; Lopez-Paredes, A. Learning in continuous double auction market. In *Artificial Economics*; Springer: Berlin/Heidelberg, Germany, 2006; pp. 41–51.
73. Hartigan, J.A.; Hartigan, P.M.; others. The dip test of unimodality. *Ann. Stat.* **1985**, *13*, 70–84.
74. Donnelly, N.; Cave, K.; Welland, M.; Menneer, T. Breast screening, chicken sexing and the search for oil: Challenges for visual cognition. *Geol. Soc. Lond. Spec. Publ.* **2006**, *254*, 43–55.
75. Nisbett, R.E.; Ross, L. *Human Inference: Strategies and Shortcomings of Social Judgment*; 1980. Available online: https://philpapers.org/rec/nishis (accessed on 10 May 2021) .
76. Dave, C.; Wolfe, K.W. On confirmation bias and deviations from Bayesian updating. *Retrieved* **2003**, *24*, 2011.
77. Nickerson, R.S. Confirmation bias: A ubiquitous phenomenon in many guises. *Rev. Gen. Psychol.* **1998**, *2*, 175–220.
78. Mataric, M.J. Designing emergent behaviors: From local interactions to collective intelligence. In Proceedings of the Second International Conference on Simulation of Adaptive Behavior, Honolulu, Hawaii, 13 April 1993; pp. 432–441.
79. Tenenbaum, J.B.; Kemp, C.; Griffiths, T.L.; Goodman, N.D. How to grow a mind: Statistics, structure, and abstraction. *Science* **2011**, *331*, 1279–1285.
80. Vul, E.; Pashler, H. Measuring the crowd within probabilistic representations within individuals. *Psychol. Sci.* **2008**, *19*, 645–647.
81. Lewandowsky, S.; Griffiths, T.L.; Kalish, M.L. The wisdom of individuals: Exploring people's knowledge about everyday events using iterated learning. *Cogn. Sci.* **2009**, *33*, 969–998.

82. Tenenbaum, J.B.; Griffiths, T.L.; Kemp, C. Theory-based Bayesian models of inductive learning and reasoning. *Trends Cogn. Sci.* **2006**, *10*, 309–318.
83. Baker, C.L.; Saxe, R.; Tenenbaum, J.B. Action understanding as inverse planning. *Cognition* **2009**, *113*, 329–349.
84. Tversky, A.; Kahneman, D. Judgment under uncertainty: Heuristics and biases. *Science* **1974**, *185*, 1124–1131.
85. Nisbett, R.E.; Kunda, Z. Perception of social distributions. *J. Personal. Soc. Psychol.* **1985**, *48*, 297.
86. Lindskog, M. Is the Intuitive Statistician Eager or Lazy?: Exploring the Cognitive Processes of Intuitive Statistical Judgments. Ph.D Thesis, Acta Universitatis Upsaliensis , 2013. Available online: https://www.diva-portal.org/smash/record.jsf?pid=diva2%3A677471&dswid=8406 (accessed on 10 May 2021)
87. Hoffman, A.B.; Rehder, B. The costs of supervised classification: The effect of learning task on conceptual flexibility. *J. Exp. Psychol. Gen.* **2010**, *139*, 319.
88. Malkiel, B.G.; Fama, E.F. Efficient capital markets: A review of theory and empirical work. *J. Financ.* **1970**, *25*, 383–417.
89. Fama, E.F. The behavior of stock-market prices. *J. Bus.* **1965**, *38*, 34–105.
90. Shanteau, J. Psychological characteristics and strategies of expert decision makers. *Acta Psychol.* **1988**, *68*, 203–215.
91. Koehler, D.J.; Brenner, L.; Griffin, D. The calibration of expert judgment: Heuristics and biases beyond the laboratory. *Heuristics Biases Psychol. Intuitive Judgm.* **2002**, 686–715. Available online: https://psycnet.apa.org/record/2003-02858-039 (accessed on 10 May 2021).
92. Lakshminarayanan, V.R.; Chen, M.K.; Santos, L.R. The evolution of decision-making under risk: Framing effects in monkey risk preferences. *J. Exp. Soc. Psychol.* **2011**, *47*, 689–693.
93. Mallpress, D.E.; Fawcett, T.W.; Houston, A.I.; McNamara, J.M. Risk attitudes in a changing environment: An evolutionary model of the fourfold pattern of risk preferences. *Psychol. Rev.* **2015**, *122*, 364.
94. Kenrick, D.T.; Griskevicius, V. *The Rational Animal: How Evolution Made Us Smarter than We Think*; Basic Books (AZ): 2013. Available online: https://psycnet.apa.org/record/2013-31943-000 (accessed on 10 May 2021)
95. Josef, A.K.; Richter, D.; Samanez-Larkin, G.R.; Wagner, G.G.; Hertwig, R.; Mata, R. Stability and change in risk-taking propensity across the adult life span. *J. Personal. Soc. Psychol.* **2016**, *111*, 430.
96. Cronqvist, H.; Siegel, S. The genetics of investment biases. *J. Financ. Econ.* **2014**, *113*, 215–234.
97. Santos, L.R.; Rosati, A.G. The evolutionary roots of human decision making. *Annu. Rev. Psychol.* **2015**, *66*, 321–347.
98. Mishra, S. Decision-making under risk: Integrating perspectives from biology, economics, and psychology. *Personal. Soc. Psychol. Rev.* **2014**, *18*, 280–307.
99. Azuma, R.; Daily, M.; Furmanski, C. A review of time critical decision making models and human cognitive processes. In Proceedings of the 2006 IEEE Aerospace Conference, Big Sky, MT, USA, 4–11 March 2006; p. 9.
100. Cohen, I. Improving time-critical decision making in life-threatening situations: Observations and insights. *Decis. Anal.* **2008**, *5*, 100–110.
101. Van Knippenberg, D.; Dahlander, L.; Haas, M.R.; George, G. *Information, Attention, and Decision Making*; 2015. Available online: https://psycnet.apa.org/record/2015-33332-001 (accessed on 10 May 2021)
102. Lubell, M.; Scholz, J.T. Cooperation, reciprocity, and the collective-action heuristic. *Am. J. Political Sci.* **2001**, pp. 160–178.
103. Rand, D.G.; Brescoll, V.L.; Everett, J.A.; Capraro, V.; Barcelo, H. Social heuristics and social roles: Intuition favors altruism for women but not for men. *J. Exp. Psychol. Gen.* **2016**, *145*, 389.
104. Limpert, E.; Stahel, W.A.; Abbt, M. Log-normal distributions across the sciences: keys and clues: on the charms of statistics, and how mechanical models resembling gambling machines offer a link to a handy way to characterize log-normal distributions, which can provide deeper insight into variability and probability—Normal or log-normal: that is the question. *BioScience* **2001**, *51*, 341–352.
105. Dehaene, S.; Izard, V.; Spelke, E.; Pica, P. Log or linear? Distinct intuitions of the number scale in Western and Amazonian indigene cultures. *Science* **2008**, *320*, 1217–1220.
106. Kao, A.B.; Berdahl, A.M.; Hartnett, A.T.; Lutz, M.J.; Bak-Coleman, J.B.; Ioannou, C.C.; Giam, X.; Couzin, I.D. Counteracting estimation bias and social influence to improve the wisdom of crowds. *J. R. Soc. Interface* **2018**, *15*, 20180130.
107. Payne, J.W.; Payne, J.W.; Bettman, J.R.; Johnson, E.J. *The Adaptive Decision Maker*; Cambridge University Press: Cambridge, UK, 1993.
108. Schneider, W.; Shiffrin, R.M. Controlled and automatic human information processing: I. Detection, search, and attention. *Psychol. Rev.* **1977**, *84*, 1.
109. Barkoczi, D.; Galesic, M. Social learning strategies modify the effect of network structure on group performance. *Nat. Commun.* **2016**, *7*, 1–8.
110. Krafft, P.M.; Della Penna, N.; Pentland, A.S. An experimental study of cryptocurrency market dynamics. In Proceedings of the 2018 CHI Conference on Human Factors in Computing Systems, Montreal, QC, Canada, 21–26 April 2018; pp. 1–13.
111. Plackett, R.L.; Burman, J.P. The design of optimum multifactorial experiments. *Biometrika* **1946**, *33*, 305–325.
112. Montgomery, D.C. *Design and Analysis of Experiments*; John Wiley & Sons: Hoboken, NJ, USA, 2017.
113. Pearl, J. Causal diagrams for empirical research. *Biometrika* **1995**, *82*, 669–688.
114. Rubin, D.B. Causal inference using potential outcomes: Design, modeling, decisions. *J. Am. Stat. Assoc.* **2005**, *100*, 322–331.
115. Alquist, R.; Kilian, L. What do we learn from the price of crude oil futures? *J. Appl. Econom.* **2010**, *25*, 539–573.
116. French, K.R. Detecting spot price forecasts in futures prices. *J. Bus.* **1986**, *59*, S39–S54.

117. Kim, Y.S.;Walls, L.A.; Krafft, P.; Hullman, J. A Bayesian Cognition Approach to Improve Data Visualization. In Proceedings of the 2019 CHI Conference on Human Factors in Computing Systems, Scotland, UK 4–9 May 2019; p. 682.
118. Vul, E.; Goodman, N.; Griffiths, T.L.; Tenenbaum, J.B. One and done? Optimal decisions from very few samples. *Cogn. Sci.* **2014**, *38*, 599–637.
119. Deshpande, S. Brexit Myth on FTSE and DAX Companies: A Review. Available at SSRN 3517139 2020. Available online: https://www.researchgate.net/profile/Shubhada-Deshpande-2/publication/338502066_Brexit_Myth_on_FTSE_and_DAX_Companies_A_Review/links/5e183584a6fdcc2837662070/Brexit-Myth-on-FTSE-and-DAX-Companies-A-Review.pdf (accessed on 10 May 2021)
120. Cox, J.; Griffith, T. Political Uncertainty and Market Liquidity: Evidence from the Brexit Referendum and the 2016 US Presidential Election. Available at SSRN 3092335. 2018. Available online: https://papers.ssrn.com/sol3/papers.cfm?abstract_id=3092335 (accessed on 10 May 2021)
